# Development and psychometric evaluation of the death risk perception scale for advanced cancer patients

**DOI:** 10.1186/s12904-024-01467-7

**Published:** 2024-05-29

**Authors:** Guojuan Chen, Xiaoling Zhang, Zhangxian Chen, Shangwang Yang, Jianwei Zheng, Huimin Xiao

**Affiliations:** 1 School of Nursing, Fujian Medical University, Fujian, China; 2 Clinical Oncology School of Fujian Medical University, Fujian Cancer Hospital, Fuzhou, China; 3 Department of Medical Oncology, Rehabilitation Hospital Affiliated to Fujian University of Traditional Chinese Medicine, Fuzhou, China; 4 Department of Oncology, The Union Hospital Affiliated with Fujian Medical University, Fuzhou, China; 5 Research Center for Nursing Humanity, Fujian Medical University, No 1 Xuefu North Road, University Town, Shangjie town, Fuzhou, Minhou County, Fujian Province China

**Keywords:** Risk perception, Advanced cancer, Palliative care, Psychometrics

## Abstract

**Background:**

An accurate perception of death risk is a prerequisite for advanced cancer patients to make informed end-of-life care decisions. However, there is to date no suitable scale to measure death risk perception. This study was to develop and psychometrically test the death risk perception scale (DRPS) for advanced cancer patients.

**Methods:**

Process of instrument development and psychometric evaluation were used. First, qualitative research, a literature review, brainstorming, a Delphi study, and cognitive interviews were conducted to construct a pretest scale of death risk perception. Second, a scale-based survey was administered to 479 advanced cancer patients. Item, exploratory factor, and confirmatory factor analyses were employed to optimize the scale. The Cronbach’s alpha was calculated as a reliability analysis. The validity analysis included construct, convergent, discriminant, and content validity values.

**Results:**

A three-dimension, 12-item scale was developed, including deliberative, affective, and experiential risk perception. The confirmatory factor analysis supported the three-factor model with satisfactory convergent and discriminant validity levels. The Cronbach's alpha coefficient for internal consistency was 0.807 and scale-level content validity index was 0.98.

**Conclusions:**

The 12-item DRPS is a reliable and valid instrument for assessing the level of death risk perception in advanced cancer patients. More studies are needed to examine its structure and robustness prior to use.

**Supplementary Information:**

The online version contains supplementary material available at 10.1186/s12904-024-01467-7.

## Background

Cancer has become a leading cause of premature mortality and decline in life expectancy in many countries, due to the increase and overall aging of the global population [1]. According to the International Agency for Research on Cancer, the disease was responsible for an estimated 10 million deaths in 2020 and will increase by 50% over the next 20 years [2]. Advanced cancer is often defined as “cancers that cannot be cured,” due to the limited availability of curative treatment options [3]. Unfortunately, many patients maintain overly optimistic beliefs regarding survival time and chances for a cure [4]. A systemic review reported that up to 75% of advanced cancer patients were unaware of their poor prognosis, and the use of aggressive treatments, mainly chemotherapy, has increased by 50% [5]. Furthermore, some patients still received life-sustaining interventions such as ventilators and artificial feeding at the end of their lives, prolonging the painful dying process and preventing them from dying in comfort and with dignity.

An accurate perception of death risk is a prerequisite for advanced cancer patients making informed decisions [6]. Risk perception involves individuals’ intuitive judgments and subjective perceptions of risk [7]. For advanced cancer patients, the looming threat of impending death is the main risk [8]. Underestimation the risk of death leads to unrealistic hopes and over-treatment, while overestimation causes anxiety, depression, and a reduced quality of life [9, 10]. Previously, researchers assessed the perception of death risk of advanced cancer patients through prognostic awareness. It involves understanding rehabilitation opportunities, limited survival time, life expectancy, treatment purpose, and disease progression [5, 11]. Tools have been developed to evaluate the level of prognostic awareness among such patients, including interview assessments, simple questionnaires, and structured scales. For example, Trevino et al., utilized an open-ended question to collect patients’ estimates of life expectancy: “How long do you think you can live?” [12] Despite this method being simple and practical, the results tended to be subjective and influenced by the researcher’s experience. Another option is a simple questionnaire. Tang et al., presented patients with advanced cancer with multiple options to choose from when assessing their perceived prognostic status [13]. Since simple questionnaires often feature one to four questions and alternative answers, they may not fully capture patients’ perceptions of their prognosis. The Prognosis and Treatment Perception Questionnaire is a structured scale commonly used to assess patients’ perception of their prognosis. Although the scale presents patients with items and various options related to prognostic awareness, it can only calculate the percentage of each option and fails to provide a quantifiable differentiation of the perception level of the prognosis [14]. More importantly, prognostic awareness is limited to objective information about the disease, whereas death risk perception also involves non-rational factors such as emotions and intuition [15]. Thus, a tool used to assess prognostic awareness cannot accurately evaluate death risk perception in advanced cancer patients.

Apart from prognostic awareness, scholars have also explored death risk perception among advanced cancer patients through the lens of death awareness. Currently, death awareness scales include the Self-Death Awareness Scale (SDAS) and Multidimensional Mortality Awareness Measure Questionnaire (MMA-Measure). The SDAS was developed by Thomes to measure awareness of mortality in healthy individuals in the US [16]. However, some items on the scale are expressed insensitively and may potentially cause patients psychological discomfort, limiting its application [17]. The MMA-Measure identifies five dimensions of death awareness: legacy, fearfulness, acceptance, disempowerment, and disengagement [18]. It was designed to explore the complex nature of human mortality awareness, focusing on both the positive and negative aspects of death attitudes [19]. Death risk perception encompasses a patient’s evaluation of their risk of death, and takes into account their experiences, knowledge, and communication with healthcare providers. Therefore, a death risk perception scale for advanced cancer patients facing death was needed. This study aimed to develop a death risk perception scale (DRPS) based on risk perception theory specifically for patients with advanced cancer, providing medical professionals with a reliable tool for evaluating their patients’ subjective understanding of death risk.

## Methods

The study adopted the scale development methodology form Robert F. DeVellis [20] and followed the COSMIN guideline [21]. It consisted of two phases. Phase 1 involved the development of the pretest version of the DRPS. Phase 2 were the refinement and psychometric evaluation of the scale through a cross-sectional survey.

### Framework

The Tripartite Model of Risk Perception (TRIRISK) proposed by Ferrer et al., [22] was used as the theoretical framework for this study. It suggests that the concept of risk perception involves three distinct components: (1) deliberative risk perception: probability judgment or deliberation based on systematic and rational thinking; (2) affective risk perception: the valence (positive vs. negative) and associated arousal (high vs. low) of affective responses to the possibility of developing a disease or illness, such as worry, anxiety, or fear; and (3) experiential risk perception: outputs of experiential processing that are holistic.

### Phase 1: Development of the pretest version of the DRPS

According to the TRIRISK, three dimensions of death risk perceptions were identified including deliberative, affective, and experiential risk perceptions. Then, 43 initial items were developed through our semi-structured interviews with 28 advanced cancer. 101 items were identified from a comprehensive literature review of Chinese and international databases. Finally, a preliminary pool of 144 items were conceptualized, comprising 31 items for deliberative dimension, 77 items for affective dimension, and 36 items for experiential dimension.

To select the item pool and and improve the content validity of the DRPS, our research team conducted a brainstorming session to initially review and discuss the reasonability and phrasing of each item. Ultimately, a set of 42 items with three dimensions was generated, with each item rated on a 5-point Likert scale from 1 = strongly disagree to 5 = strongly agree. Then, a Delphi survey was conducted, involving 21 experts invited from 11 provinces across China. They all held a senior professional title and had expertise in thanatology, palliative care, oncology medicine/nursing, medical humanities, or psychometrics. The panelists rated the importance of each item and dimension using a 5-point Likert scale (ranging from 1 = not important to 5 = very important) and provided comments and suggestions for additional items, based on their experience and knowledge. Criteria for screening items was a coefficient of variation (CV) of ≥.25 [23]. Two rounds of surveys were conducted to test the content validity of the scale. In the first round, five items were more than 0.25 and were considered for deletion. 15 items were deleted, 18 items were modified, and two were added, as suggested by expert opinions and discussion results. In the second round, all items met the criteria, two were added, and two were modified after discussion by the research team. Finally, the DRPS had a total of 27 items and three dimensions.

To test whether the statement of the items was clear enough for reading and answering, two rounds of cognitive interviews were conducted, with 15 advanced cancer patients taking part in each round. In the first round, eight respondents recommended to remove any terms related to death and four suggested the Likert scale changed from 5 to 7 points to improve the clarity of the instructions. In the second round, 15 respondents did not have further questions and the preliminary scale with three dimensions and 27 items was formulated using a 7-point Likert scale (Appendix 1).

### Phase 2: Refinement and psychometric evaluation of the DRPS

#### Participants

A cross-sectional study was conducted from December 2022 to February 2023. Convenience sampling was used to recruit patients from the inpatient cancer departments of three tertiary hospitals in Fujian Province, China. The inclusion criteria were as follows: (1) diagnosed with advanced cancer at Stages III or IV, (2) aged 18 years or above, and (3) aware of their diagnosis, disease conditions, and treatment. The exclusion criteria were as follows: (1) severely disabled or critically ill (Karnofsky Performance Status (KPS) < 20%), (2) verbal communication or cognitive impairment, or (3) psychiatric disorder.

#### Sample size

The sample size was calculated based on a range of five to 10 participants per item [21]. Because 27 items were presented in the primary scale, a sample size of 108 to 270 participants was recommended. Considering a 10% rate of invalid scales, the estimated sample size increased to 119 to 297 patients. Finally, the total valid scales collected was 479, with a response rate of 98.76% (479/485). Given that the sample size for confirmatory factor analysis (CFA) should be larger than for exploratory factor analysis (EFA), we utilized 230 samples for the item and exploratory factor analyses and 249 samples for the confirmatory factor, validity, and reliability analyses.

#### Instruments

##### Personal information form

Socio-demographic data were collected, including age, gender, marital status, income level, education level, and religious beliefs, as well as information related to the patient’s disease diagnosis, stage, course, and treatment.

##### Death risk perception scale (primary version)

The primary version of DRPS was used to assess the level of death risk perception of advanced cancer patients. There were 27 items, with each item rated on a 7-point Likert scale ranging from 1 = very much disagree to 7 = very much agree. The higher composite scores represented greater perceptions of death risk.

### Data collection

Before the study commenced, approval was obtained from the corresponding author’s university and study setting. Data were collected by two trained research assistants, who also introduced the study and invited eligible participants to fill in the scale (with informed consent). If there were difficulties in completing the scale, the assistants would read each item aloud and then objectively write down their oral response. After the investigation, assistants reviewed and verified the scale for completeness and accuracy. Any missing or incorrect information was promptly addressed with the participants.

### Data analysis

Data input, processing, and statistical analysis were performed using IBM SPSS version 26.0. The CFA was conducted using AMOS 26.0. The continuous variables were presented as mean ± standard deviation (SD), and category variables were expressed using frequency and percentage. Items were screened and revised according to the following rules [23, 24]: (1) a* t*-test was conducted for patients with total scores in the top and bottom 27%, and the results showed that *P* was > .05; (2) the correlation coefficient between the total score and each item score was < .4; (3) the Cronbach’s α coefficient decreased if one item was removed; and (4) the factor loading was < .4, or the difference between the factor loadings was < .05. An EFA using principal component analysis (PCA) with varimax rotation was conducted to examine the factor structure. The suitability of data for the PCA was assessed using the Kaiser-Meyer-Olkin (KMO) test and Bartlett's test of sphericity [25]. Subsequently, the factor structure derived from the prior EFA was tested with CFA, using the diagonally weighted least square estimator. The following indices and cut-off criteria were used to assess the model fit of the CFA model [26]: χ^2^/degree of freedom (χ^2^/*df* < 3), goodness-of-fit index (GFI > 0.9), root mean square error of approximation (RMSEA < 0.08), comparative fit index (CFI > 0.95), normed fit index (NFI > 0.9), and Tucker-Lewis index (TLI > 0.9).

Validity analysis included convergent, discriminant, and content validity. Convergent analysis was assessed through factor loading, average variance extracted (AVE), and composite reliability (CR). Generally, a factor loading of greater than 0.45 is considered acceptable; an AVE higher than 0.5 and CR greater than 0.6 are also deemed acceptable [27]. If the AVE square root value was greater than the correlation coefficient, then the scale was considered to have good discriminatory validity [24]. The second Delphi survey was used to evaluate the content validity of the scale, employing the item-content validity index (I-CVI) for individual items and scale-level CVI (S-CVI) for the entire scale. The 4-point Likert scale (1 = irrelevant to 4 = very relevant) was used and the content validity index calculated by the percentage of items rated as ‘relevant’ or ‘very relevant.’ Generally, a scale with an I-CVI greater than 0.78 and S-CVI greater than 0.90 is considered to possess good content validity. The internal consistency reliability of the scale was examined using Cronbach's alpha, with an acceptable cut-off value of ≥ 0.70 for the overall scale [28].

### Ethical considerations

The present study was approved by the Ethics Committee of the corresponding author’s university. Informed consent was obtained by patients after the study’s purpose, content, and method were explained. The patients were free to withdraw at any time. All information related to the privacy of the patients was anonymized and confidential.

## Results

### Participants

The participants’ characteristics are shown in Table 1. A total of 479 patients with advanced cancer were enrolled from General Hospital A (*n* = 228), General Hospital B (*n* = 46), and Oncology Hospital (*n* = 205). The mean age was 55.25 years (SD = 11.34), and the majority were male (295/479, 61.6%), married (450/479, 93.9%), and affiliated with a religion (360/479, 75.2%). Most patients were diagnosed with digestive system cancer (305/479, 63.7%) in Stage IV (271/479, 56.6%), and the average KPS was more than 80% (399/479, 83.3%).

**Table 1:** Participant Characteristics (*n* = 479)

**Variable**	**Category**	**n (%)**
**Gender**	Male	295(61.60)
	Female	184(38.40)
**Age**	18-59	315(65.80)
	≥ 60	164(34.20)
**Marital status**	Married	450(93.90)
	Unmarried/widowed /divorced/separated	29(6.10)
**Education level**	Little or no literacy	26(5.40)
	Primary school	143(29.90)
	Junior middle school	114(23.80)
	Senior high school	113(23.60)
	University or above	83(17.30)
**Religion**	Yes	360(75.20)
	No	119(24.80)
**Employment status**	Yes	239(49.90)
	No	240(50.10)
**Treatment decision maker**	Yes	423(88.30)
	No	56(11.70)
**Health insurance**	Employee health insurance	214(44.70)
	Resident health insurance	101(21.10)
	New rural cooperative medical system	151(31.50)
	Private expense	13(2.70)
**Monthly household income (RMB)**	<1,000	45(9.40)
	1,000~3,000	157(32.80)
	3,001~6,000	129(26.90)
	> 6,000	148(30.90)
**Death experience**	Yes	333(69.50)
	No	146(30.50)
**Death education experience**	Yes	80(16.70)
	No	399(83.30)
**Type of tumor**	Digestive tumor	305(63.70)
	Respiratory tumor	66(13.80)
	Urinary tumor	6(1.30)
	Reproductive tumor	87(18.20)
	Other	15(3.00)
**Stage of tumor**	III	208(43.40)
	IV	271(56.60)
**Disease course (months)**	≤ 12	246(51.40)
	13-24	103(21.50)
	25-36	59(12.30)
	≥ 37	71(14.80)
**Treatment**	Radiotherapy/chemotherapy	271(56.60)
	No radiotherapy/chemotherapy	208(43.40)
**KPS**	≥ 80	399(83.30)
	50-70	60(12.50)
	≤ 40	20(4.20)

### Item analysis

The item analysis results indicated that 25 out of 27 items successfully met the criteria, two items (Items 20 and 26) failed to meet the requirements and were removed from the initial scale (see Appendix 2). Therefore, the scale had 25 items in total.

## Validity analysis

### Construct validity

#### Exploratory factor analysis

The KMO measure of sampling adequacy (0.859) and Bartlett’s sphericity test (*P* < .001) results implied that the data were appropriate for an EFA. All items had a 0.4 or greater loading value, and six factors with eigenvalues greater than 1 were extracted. However, the scree plot showed that the slope became relatively flat after the third factor. Based on the TRIRISK, we set the number of common factors to 3 and conducted a second PCA and varimax rotation. The analysis revealed that Item 13 had double the loadings on Factors 1 and 3, with little variation in loading amount. Additionally, Items 7, 8, 9, 10, 11, 12, 13, 17, 25, and 27 differed from the attribution dimensions established in this study. We applied the screening rules to select the items and verified the results through several rounds of EFA. Finally, a three-dimensional 12-item measure was presented as the DRPS, accounting for 69.252% of the total variance. The three factors were named: (1) deliberative risk perception, (2) affective risk perception, and (3) experiential risk perception (see Table 2).

**Table 2:** Rotated Factor Loadings for the 12-Item death risk perception questionnaire (*n* = 230)

**Number**	**Item**	**Factor loading**			**Communality**
		**Factor 1:** **Deliberative** **risk perception**	**Factor 2:** **Affective risk perception**	**Factor 3:** **Experiential risk perception**	
5	The recurrence of cancer makes me feel like my life is in grave danger.	0.855			0.760
6	The metastasis of my cancer makes me feel like my life is threatened.	0.817			0.722
1	Cancer diagnosis makes me feel like my life is at risk.	0.795			0.656
2	Abnormal indicators make me feel anxious about my life.	0.781			0.622
4	Poor treatment makes me feel like I am running out of time.	0.759			0.637
18	I try to avoid considering the possible negative consequences of the disease.			0.752	0.635
14	I can face the possible consequences of the disease with calmness and composure.			0.741	0.573
16	I am afraid of the potential harmful effects of the disease.			0.710	0.766
15	The possibility of negative consequences from the disease causes me significant distress.			0.698	0.718
21	I believe spiritual strength can assist me in overcoming my disease.		0.892		0.799
23	I believe that as long as we deal with the disease, there is hope for life.		0.864		0.752
19	I believe modern medical technology can control my disease.		0.817		0.669

### Confirmatory factor analysis

The 12-item three-factor structure found with EFA was tested with CFA. The fit indices were all acceptable and demonstrated a good fit for the three-factor structure: χ^2^/*df* = 2.376, GFI = 0.926, CFI = 0.953, IFI = 0.973, NFI = 0.922, RMSEA = 0.078, and TLI = 0.935.

## Convergent and discriminant validity

Regarding convergent validity, the factor loadings of all items were greater than 0.45, except for Item 18. Considering that Item 18 and others (14, 15, and 16) measured the affective risk perception dimension, including factors such as escape, acceptance, distress, and fear, it was retained after discussion with the research team. The AVE values for the 12 items were above 0.5, and the CR values of the three dimensions were all over 0.70. AVE square root values were greater than correlations between factors of the scale (see Appendix 3). The convergent and discriminant validity results of the scale were above the acceptable value.

### Content validity

The results showed that the I-CVI values of the 12 items ranged from 0.947 to 1.000, and the S-CVI value was .98 (see Appendix 4).

### Reliability analysis

The Cronbach's α coefficient for the DRPS was 0.807. The internal consistency values for the three domains were .878, .775, and .829.

### Final instrument

Based on the results of the item, reliability, and validity analyses, the death risk perception scale for patients with advanced cancer consisted of 12 items in three dimensions, deliberative risk perception (five items), affective risk perception (four items), and experiential risk perception (three items). Items were measured using a 7-point Likert scale ranging from 1 (strongly disagree) to 7 (strongly agree). The total score was calculated by summing up the scores of all the items, with a higher total score indicating a higher perception of death risk for the patient.

## Discussion

This study developed a tool for evaluating the death risk perception of advanced cancer patients and assessed that tool’s psychometric properties. The 12-item DRPS was developed based on a theoretical framework, qualitative research, a literature review, brainstorming, a Delphi study, cognitive interviews, and field investigation. The results of the psychometric properties testing provided initial evidence of the instrument’s validity and reliability, making it a valuable tool for clinical and research purposes related to assessing advanced cancer patients’ levels of death risk perception.

The dimensions of the DRPS covered various aspects of risk perception, including deliberative, affective, and experiential risk perception. Generally, deliberative risk perception has been the primary focus of risk perception research. Previous studies [29, 30] have indicated that patients’ perceptions of death risk depended on expert communication between doctors and patients regarding objective aspects of the disease such as disease diagnosis, progression, and treatment purpose. Some qualitative studies [31, 32] have also discovered that death risk perception is also influenced by the information provided in medical examination report including texts, images, and other relevant data, as collectively reflected in the items of the DRPS.personal emotions are also integral components of risk perception [33, 34]. Some scholars [35, 36] have proposed that individuals with higher negative emotional reactivity tend to have a higher risk perception of dangerous things. In many cultures, advanced cancer is regarded as a poignant reminder of mortality, associated with bad luck and evoking negative emotions [37]. Therefore, when confronted with the looming threat of death, these patients may perceive a greater risk. However, patients with advanced cancer who exhibit a peaceful emotional response and are willing to confront their own mortality often experience a diminished perception of the risk of death.

Individual risk perception is also influenced by past experiences and cognitive judgments. Studies have shown that factors like trust in doctors and confidence in treatment effectiveness can impact risk perception [38–40]. Some qualitative studies [31, 41] have revealed that some advanced patients with cancer believe that it inevitably leads to death, resulting in a high level of death risk perception, even if treatment is effective. However, other patients have confidence in medical technology and trust in doctors, leading to a lower perception of the risk of death. The overall high perception of death risk is associated with negative psychological consequences such as anxiety, depression, and a decreased quality of life. Conversely, a lower perception of death risk can lead to unrealistic hope and over-treatment, which may not yield favorable clinical outcomes for patients.

The validity of the DRPS was found to be good. First, the three factors contributed to a cumulative variance of 69.252%, which exceeded the acceptable threshold of 60%. Besides, the results of the CFA indicated that both the absolute and value-added fit indices met the standard, and the model structure was found to be stable. As shown in the convergent validity, the factor loadings and AVE were greater than 0.5, and the CR was greater than 0.6, indicating good convergent validity of the scale. A discriminant validity analysis revealed that the square root of the AVE value of each dimension was greater than the correlation coefficient between one dimension and the others, suggesting that the inter-dimensional validity was acceptable. As for content validity, the results showed that the I-CVI values for the 12 items ranged from 0.947 to 1.000, with all values exceeding 0.78. The S-CVI value was 0.982, greater than 0.90, which indicated the good content validity of the scale. Overall, the validity of the developed DRPS were all relatively good or acceptable, suggesting its efficacy in effectively assessing the level of death risk perception in patients with advanced cancer.

The Cronbach's α coefficient was used in this study to determine intrinsic consistency. The results showed a total Cronbach's α coefficient of 0.807 and all three dimensions exceeded α > 0.70, indicating the good intrinsic consistency of the DPRS. Moreover, the scale is feasible and practical for measuring death risk perception. In this study, 485 scales were distributed and 479 valid responses obtained, resulting in a scale recovery rate of 98.76% and indicating a high level of acceptance of the scale [20]. Furthermore, the final DRPS was found to take less than 10 minutes to complete, imposing minimal physical and psychological burden on patients with advanced cancer. The scale effectively assessed the perceived risk of death in these patients and can be applied in future evaluations.

It is crucial to assess the patients’ death risk perception, particularly when their condition changes or medical treatment requires replacement. The DRPS is a comprehensive tool for clinical staff to assess patients’ perception of death risk from three distinct components: deliberative, affective, and experiential risk perception. It has the potential to be invaluable in understanding how risk perception influences risk occurrence and behavioral decision making. Additionally, this scale can provide theoretical references and practical evidence for specific and targeted interventions. By comparing patients’ scores in each dimension with their actual disease condition, clinical staff can identify discrepancies in death risk perception and provide appropriate guidance. This may involve providing a disease prognosis, enhancing emotional well-being, and revising cognition of disease to achieve a reasonable perception of risk.

### Limitations and implication

This study has several limitations. First, due to the physical instability and condition changes of the respondents, test-retest reliability was unsuitable. Second, this study used a high proportion of male respondents and participants with a junior high school level of education or below. It is essential to note that individuals who refused or lacked the physical or psychological ability to participate may have possessed unique characteristics, leading to a potential bias in the sample. Third,the participants were recruited from three hospitals in one city, which may limit the representativeness of the findings to advanced cancer patients in other regions. Therefore, future studies should evaluate the cross-cultural applicability of the DRPS to ensure its psychometric properties internationally. Additionally, we did not find a suitable scale for assessing the convergent validity and discriminant validity of the DRPS based on the systematic review. Future research can more thoroughly examine the scale’s convergent and discriminant validity by comparing it with similar assessment scales.

## Conclusions

To the best of our knowledge, the DRPS is the first scale developed to assess the risk perception of death in advanced cancer patients. Our study provides evidence that the DRPS is a promising tool with satisfactory reliability and validity. More studies are needed to examine its clinical utility, both nationally and internationally.

### Supplementary Information

Supplementary Material 1.

## Data Availability

The datasets used and/or analyzed during the current study are available from the corresponding author on reasonable request.

## Abbreviations

DRPS: Death risk perception scale
SDAS: Self-Death Awareness Scale
MMA-Measure: Multidimensional Mortality Awareness Measure Questionnaire
TRIRISK: Tripartite Model of Risk Perception
CV: Coefficient of variation
KPS: Karnofsky Performance Status
CFA: Confirmatory factor analysis
EFA: Exploratory factor analysis
PCA: Principal component analysis
KMO: Kaiser-Meyer-Olkin
GFI: Goodness-of-fit index
CFI: Comparative fit index
