# Correction: Development and psychometric evaluation of the death risk perception scale for advanced cancer patients

**DOI:** 10.1186/s12904-024-01491-7

**Published:** 2024-06-25

**Authors:** Guojuan Chen, Xiaoling Zhang, Zhangxian Chen, Shangwang Yang, Jianwei Zheng, Huimin Xiao

**Affiliations:** 1 School of Nursing, Fujian Medical University, Fujian, China; 2 Clinical Oncology School of Fujian Medical University, Fujian Cancer Hospital, Fuzhou, China; 3 Department of Medical Oncology, Rehabilitation Hospital Affiliated to Fujian University of Traditional Chinese Medicine, Fuzhou, China; 4 Department of Oncology, The Union Hospital Affiliated with Fujian Medical University, Fuzhou, China; 5 Research Center for Nursing Humanity, Fujian Medical University, No 1 Xuefu North Road, University Town, Shangjie town, Fuzhou, Minhou County, Fujian Province China

**Correction: BMC Palliat Care 23, 136 (2024).**

10.1186/s12904-024-01467-7.

Following publication of the original article [1], the authors reported that the equal contribution for the first and second author was not stated. It should be included as follows: Guojuan Chen and Xiaoling Zhang are co-first authors.

This has been included in this correction and the original article has been updated.
