# Supplementary material for: Development and psychometric evaluation of the death risk perception scale for advanced cancer patients
Source: BMC Palliat Care. 2024 May 29;23:136. doi: 10.1186/s12904-024-01467-7 (PMC11134621; doi:10.1186/s12904-024-01467-7)
Supplement: Supplementary file 1 — Supplementary Material 1. [file 12904_2024_1467_MOESM1_ESM.doc]

**Appendix 1 Primary version of the Death Risk Perception Scale**

|  | |
| --- | --- |
| **Dimension** | **Items** |
| **Deliberative**  **risk perception** | 1.Cancer diagnosis makes me feel like my life is at risk. |
|  | 2.Abnormal indicators make me feel anxious about my life. |
|  | 3.The treatment solely aims to alleviate the symptoms that give me a sense of impending danger to my life. |
|  | 4.Poor treatment makes me feel like I am running out of time. |
|  | 5.The recurrence of cancer makes me feel like my life is in grave danger. |
|  | 6.The metastasis of my cancer makes me feel like my life is threatened. |
| **Affective**  **risk perception** | 7.A cancer diagnosis prompts me to think of my own personal life. |
|  | 8.Abnormal physical symptoms prompt me to think of my personal life. |
|  | 9.Going to the hospital prompts me to think of my personal life. |
|  | 10.My family's "unusual" behavior prompts me to think of my personal life. |
|  | 11.The medical staff's "abnormal" behavior prompts me to think of my personal life. |
|  | 12.When others discuss my illness, it concerns me regarding personal matters in my life. |
|  | 13.When others discuss the topic of death, it concerns me regarding personal matters in my life. |
|  | 14.I can face the possible consequences of the disease with calmness and composure. |
|  | 15.The possibility of negative consequences from the disease causes me significant distress. |
|  | 16.I am afraid of the potential harmful effects of the disease. |
|  | 17.I hesitate to make any future plans due to my illness. |
|  | 18.I try to avoid considering the possible negative consequences of the disease. |
| **Experiential**  **risk perception** | 19.I believe modern medical technology can control my disease. |
|  | 20.I believe I have just enough money to control my disease. |
|  | 21.I believe spiritual strength can assist me in overcoming my disease. |
|  | 22.I feel like death could happen to me at any time. |
|  | 23.I believe that as long as we deal with the disease, there is hope for life. |
|  | 24.I believed I would outlive others with the same disease. |
|  | 25.Experiencing similar symptoms as a dying patient makes me feel threatened. |
|  | 26.Seeing my fellow patients’ positive treatment outcomes give me hope for effectively managing my own illness. |
|  | 27.Poor health feels like a constant threat to my life. |

**Appendix 2 Death risk perception scale: item analysis**

|  | | | | |
| --- | --- | --- | --- | --- |
| **Items** | **Critical ratio** | | **Item-total correlation** | **Cronbach’s α if item deleted** |
|  | ***t*** | ***P*** |  |  |
| 1 | -9.414 | <0.001 | 0.629 | 0.877 |
| 2 | -10.911 | <0.001 | 0.679 | 0.876 |
| 3 | -9.250 | <0.001 | 0.597 | 0.878 |
| 4 | -11.731 | <0.001 | 0.674 | 0.876 |
| 5 | -11.826 | <0.001 | 0.693 | 0.876 |
| 6 | -10.515 | <0.001 | 0.672 | 0.877 |
| 7 | -11.476 | <0.001 | 0.670 | 0.877 |
| 8 | -8.387 | <0.001 | 0.568 | 0.879 |
| 9 | -9.398 | <0.001 | 0.600 | 0.878 |
| 10 | -6.317 | <0.001 | 0.531 | 0.880 |
| 11 | -7.312 | <0.001 | 0.594 | 0.878 |
| 12 | -9.658 | <0.001 | 0.604 | 0.878 |
| 13 | -11.403 | <0.001 | 0.690 | 0.875 |
| 14 | -6.336 | <0.001 | 0.401 | 0.885 |
| 15 | -11.342 | <0.001 | 0.700 | 0.875 |
| 16 | -11.173 | <0.001 | 0.693 | 0.876 |
| 17 | -10.928 | <0.001 | 0.583 | 0.879 |
| 18 | -3.667 | <0.001 | **0.291** | 0.887 |
| 19 | -4.799 | <0.001 | **0.316** | 0.885 |
| 20 | **-0.412** | **0.681** | **-0.014** | **0.894** |
| 21 | -4.286 | <0.001 | **0.262** | 0.886 |
| 22 | -4.294 | <0.001 | **0.313** | 0.886 |
| 23 | -4.383 | <0.001 | **0.237** | 0.886 |
| 24 | -4.254 | <0.001 | **0.328** | 0.884 |
| 25 | -8.077 | <0.001 | 0.532 | 0.880 |
| 26 | **-1.881** | **0.063** | **0.177** | 0.887 |
| 27 | -8.420 | <0.001 | 0.583 | 0.879 |

**Note：**After comparing the means of each item between the high and low groups, Items 20 and 26 showed no statistical significance (t = -0.412, p = .681; t = -1.881, p = .063) and were deleted. The results of the analysis of the item-total correlation coefficients showed that eight items needed to be deleted due to their low correlations with the total scores (0.4 or lower). These were Items 18, 19, 20, 21, 22, 23, 24, and 26. The internal consistency reliability analysis indicated that the deletion of Item 20 significantly increased the reliability coefficient of the entire scale (Cronbach's α = 0.885); thus, it was removed. After this procedure, Items 20 and 26 were determined to remove from the scale.

**Appendix 3 Convergent and Discriminant Validity Values (n=249)**

|  | **Convergent validity** | | |  | **Discriminant validity** | | |
| --- | --- | --- | --- | --- | --- | --- | --- |
|  | **Factor loadings** | **AVE** | **CR** |  | **Deliberative**  **risk perception** | **Affective**  **risk perception** | **Experiential risk perception** |
| **Deliberative**  **risk perception** | 0.589~0.918 | 0.574 | 0.867 |  | **0.758** |  |  |
| **Affective**  **risk perception** | 0.445~0.921 | 0.509 | 0.793 |  | 0.631 | **0.713** |  |
| **Experiential**  **risk perception** | 0.678~0.868 | 0.631 | 0.835 |  | -0.033 | 0.042 | **0.794** |

**Appendix 4 Content validity of each item of death risk perception scale (n=249)**

| **Item** | **I-CVI** | **Item** | **I-CVI** |
| --- | --- | --- | --- |
| 1 | 1.000 | 15 | 0.947 |
| 2 | 0.947 | 16 | 1.000 |
| 4 | 1.000 | 18 | 1.000 |
| 5 | 1.000 | 19 | 1.000 |
| 6 | 1.000 | 21 | 0.947 |
| 14 | 0.947 | 23 | 1.000 |

Abbreviations: I-CVI, item-content validity index
